# Pulmonary Thromboendarterectomy Without Circulatory Arrest

**DOI:** 10.21470/1678-9741-2020-0534

**Published:** 2022

**Authors:** Reuben Lamiaki Kynta, Sanjib Rawat, Mrinal Mandal, Manuj Kumar Saikia

**Affiliations:** 1 Department of Cardiothoracic and Vascular Surgery, North Eastern Indira Gandhi Regional Institute of Health and Medical Sciences, Shillong, Meghalaya, India.

**Keywords:** Pulmonary Hypertension, Pulmonary Artery, Endarterectomy, Cardiopulmonary Bypass, Temperature, Oxygen, Tricuspid Valve Insufficiency, Quality of Life

## Abstract

**Introduction:**

Here we describe our technique and results of beating heart pulmonary thromboendarterectomy (PTE) with cardiopulmonary bypass (CPB) in four patients for treatment of chronic thromboembolic pulmonary hypertension (CTEPH).

**Methods:**

Retrospective analysis of data from patients who underwent PTE for CTEPH between January 2019 and September 2020. Patients were followed up with clinical assessment, 2D echocardiography, and computed tomography pulmonary angiogram.

**Results:**

Four patients were operated for CTEPH using our technique. Moderate tricuspid regurgitation (TR) and severe TR were found in two patients each. Severe right ventricular (RV) dysfunction was found in all cases. Thrombi were classified as Jamieson type II in three cases and type I in one case. Postoperative median direct manometric pulmonary artery (PA) pressures decreased (from 46.5 mmHg to 23.5 mmHg), median CPB time was 126 minutes, and median temperature was 33.35 °C. Mechanical ventilation was for a median of 19.5 hours. There was one re-exploration. Median intensive care unit stay was 7.5 days. There was no mortality. Postoperative 2D echocardiography revealed decrease in median PA systolic pressures (from 85 mmHg to 33 mmHg), improvement in RV function by tricuspid annular plane systolic excursion (median 14 mm *vs*. 16 mm), and improved postoperative oxygen saturations (88.5% *vs*. 99%). In follow-up (ranging between 2-15 months), all patients reported improvement in quality of life and were in New York Heart Association class I.

**Conclusion:**

With our described simple modifications, advances in perfusion, and blood conservation technologies, one can avoid the need for deep hypothermic circulatory arrest during PTE.

**Table:** 

Abbreviations, acronyms & symbols			
2D echo	= 2D echocardiography	mPAP	= Mean pulmonary artery pressure
ABP	= Arterial blood pressure	NYHA	= New York Heart Association Functional Classification
CPB	= Cardiopulmonary bypass	PA	= Pulmonary artery
CTEPH	= Chronic thromboembolic pulmonary hypertension	PAP	= Pulmonary artery pressure
CTPA	= Computed tomography pulmonary angiogram	PASP	= Pulmonary artery systolic pressure
CVP	= Central venous pressure	PTE	= Pulmonary thromboendarterectomy
DHCA	= Deep hypothermic circulatory arrest	RPA	= Right pulmonary artery
DVP	= Deep venous thrombosis	RSPV	= Right superior pulmonary vein
EF	= Ejection fraction	RV	= Right ventricular
ICU	= Intensive care unit	SpO_2_	= Oxygen saturation
IVC	= Inferior vena cava	SVC	= Superior vena cava
LPA	= Left pulmonary artery	TAPSE	= Tricuspid annular plane systolic excursion
MAP	= Mean arterial pressure	TR	= Tricuspid regurgitation
MPA	= Main pulmonary artery	UCSD	= University of California San Diego

## INTRODUCTION

Pulmonary thromboendarterectomy (PTE) is the definitive surgical treatment for chronic thromboembolic pulmonary hypertension (CTEPH)^[1]^. In this technique, a bloodless field is essential, and deep hypothermic circulatory arrest (DHCA) has been the preferred method to extract the pulmonary thrombus beyond the sublobar levels. However, DHCA is not devoid of its adverse effects which mainly include neurologic deficits^[2,3]^, renal dysfunction^[4,5]^, and bleeding^[6]^. In pursuit to control the troublesome back bleeding from the bronchial vessels and collaterals without the use of DHCA, several methods have been described to achieve complete thromboendarterectomy. Here, we describe our technique and modifications needed for PTE on beating heart cardiopulmonary bypass (CPB) with mild hypothermia on four patients.

## METHODS

### Study Design

As this was a retrospective case series, ethical clearance was waived off. Retrospective data of patients who underwent beating heart PTE from January 2019 to September 2020 at the Department of Cardiothoracic and Vascular Surgery, North Eastern Indira Gandhi Regional Institute of Health and Medical Sciences (Shillong, India), were collected, and the operative records were reviewed. This study included four patients. Written and informed consent was taken. The patients were followed up with clinical assessment, investigations like transthoracic 2D echocardiography (2D echo) and computed tomography pulmonary angiogram (CTPA) were performed, and the data was collected.

Inclusion criteria were patients reporting to/referred to our department with: (a) New York Heart Association Functional Classification (NYHA) class III & IV symptoms, (b) thrombus in main, lobar, or segmental pulmonary arteries (PA) as seen in CTPA, (c) no debilitating comorbidities, and (d) 2D echo evidence of right ventricular (RV) dysfunction. All patients with acute pulmonary embolism did not fit to undergo CPB, and those for emergency pulmonary embolectomy were excluded.

Demographic variables, pulmonary artery pressure (PAP) (systolic, diastolic, and mean), RV dysfunction as measured by tricuspid annular plane systolic excursion (TAPSE) on 2D echo, central venous pressure (CVP), oxygen saturation (SpO_2_), CPB time, location of thrombus as described by Jamieson’s classification as well as University of California San Diego (UCSD) surgical classification, postoperative course, and any postoperative complications were studied.

Pulmonary artery systolic pressure (PASP) was measured by pre- and postoperative echocardiogram. PAP was measured intraoperatively before and after PTE by direct needle manometry of main pulmonary artery (MPA). CVP and SpO_2_ were measured before and after PTE.

### Anesthetic Management

Anesthesia was induced by intravenous administration of morphine, fentanyl, and vecuronium and maintained with intravenously administered propofol and inhalation of isoflurane. Monitoring was done by a radial artery line, central venous line, electrocardiography, nasopharyngeal and rectal thermometer, and transesophageal echocardiography. The heart rate was regulated to 60-70 beats per minute by boluses of beta blocker metoprolol.

### CPB Pump and Autologous Blood Conservation Setup

**Fig. 1:**
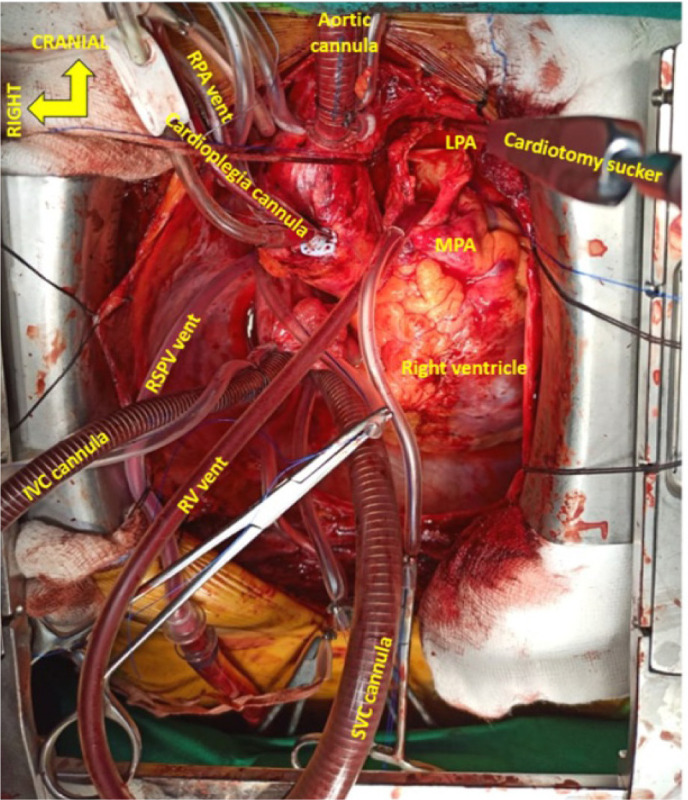
Cannulae and vent setup. Main pulmonary artery (MPA) arteriotomy extending to left pulmonary artery (LPA) with cardiotomy sucker in LPA shown. IVC=inferior vena cava; RPA=right pulmonary artery; RV=right ventricular; RSPV=right superior pulmonary vein; SVC=superior vena cava

**Fig. 2:**
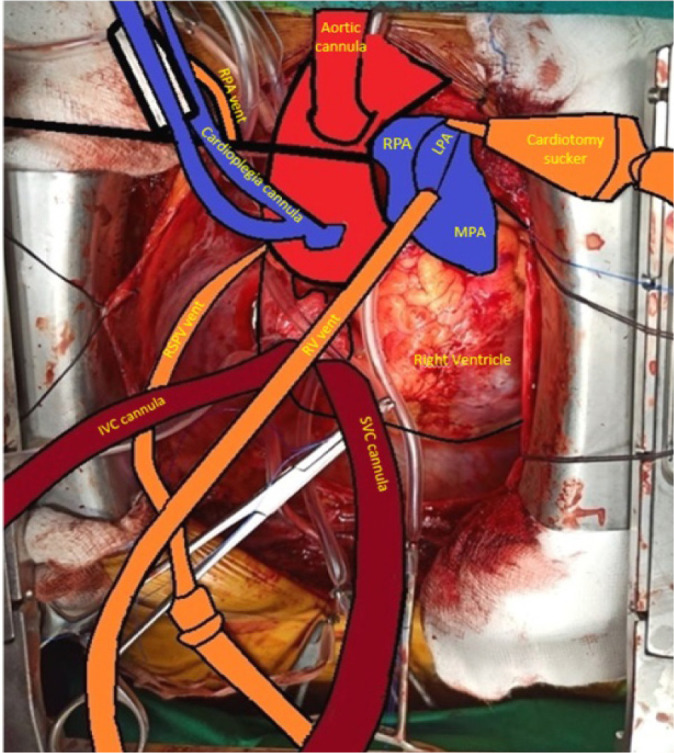
Cartoon of cannulae and vent setup. IVC=inferior vena cava; LPA=left pulmonary artery; MPA=main pulmonary artery; RPA=right pulmonary artery; RV=right ventricular; RSPV=right superior pulmonary vein; SVC=superior vena cava

Standard CPB circuit with St. Thomas II cardioplegia on standby and a cell saver (C.A.T.S®, Fresenius Kabi) is established. An aortic cannula and two straight venous cannulae for bicaval cannulation of appropriate size are used. Four pump suction lines are readied for a free cardiotomy sucker, vent sucker of the right superior pulmonary vein (RSPV), right ventricle, and either of the PAs (Figures 1 and 2). A hemoconcentrator for ultrafiltration is connected to the bypass circuit to achieve negative fluid balance. Core temperature is decreased to 32-34 °C (mild hypothermia), and flows are maintained at 2 to 2.4 l/min/m^2^. A high-pressure external sucker attached to the cell saver is used to connect an olive tip sucker, which is used as a dissector. The α-stat strategy of pH management is employed.

### Instruments Required

**Fig. 3:**
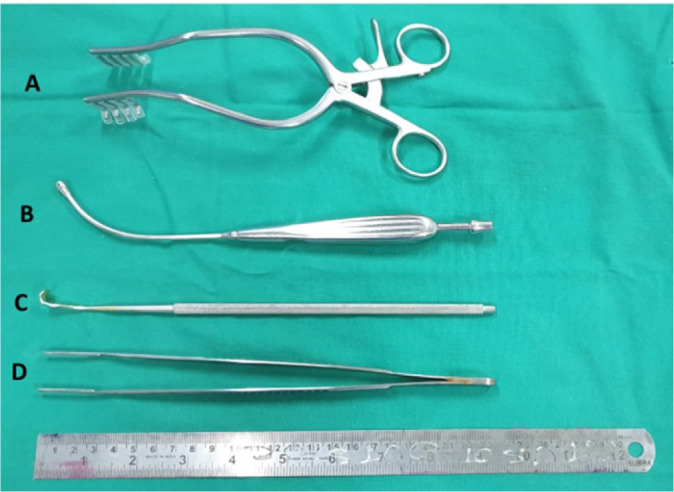
Instruments required: A) Mastoid retractor with rubber inserts on blade tips; B) Olive tip sucker; C) Ventricular septal defect retractors (23 cm length); D) DeBakey vascular forceps (23 cm length).

Two DeBakey vascular forceps (23 cm length), two ventricular septal defect retractors (23 cm length), one olive tip sucker connected to cell saver, a mastoid retractor with rubber inserts on blade tips, and a vascular clamp (straight or angled) are required (Figure 3).

### Surgical Technique

The operation is performed via a median sternotomy. After an inverted T pericardiotomy and attachment to the wound edges, direct pressure manometry by puncturing MPA is done. The superior vena cava (SVC) is looped and mobilized by cautery dissection. Mobilization of the branch PA is done intrapericardially, and none of the pleurae are opened. The aorta is looped with a vessel loop for better visualization (Figures 1 and 2).

CPB is established by cannulating the ascending aorta and both caval veins after achieving an activated clotting time of > 480 seconds with heparinization (300 U/kg).

A straight cannula in the SVC is guided high, near to the convergence of the innominate vein via the right atrial appendage, and snared. This method of cannulation further helps in proximal mobilization of the SVC. The inferior vena cava is cannulated and snared. An antegrade aortic root cardioplegia needle is inserted should the need for DHCA arise.

A vent catheter is introduced into the RSPV. The left pulmonary artery (LPA) is then dissected beyond the origin of the upper lobe artery with careful preservation of the left phrenic nerve. A small arteriotomy is made in the right pulmonary artery (RPA) just right to the aorta, and a vent catheter is introduced into it and snared. Ventilation is then stopped.

With the surgeon standing on the right side of the patient, an incision is then made in the MPA and is extended into the LPA beyond the origin of the upper lobe artery. Stay sutures are taken and the arteriotomy is exposed. A sump sucker is introduced into the right ventricle via the pulmonary valve and snared through the MPA wall. If bleeding from the RPA obscure the field of view, a vascular clamp may be applied upstream to the RPA vent.

**Fig. 4:**
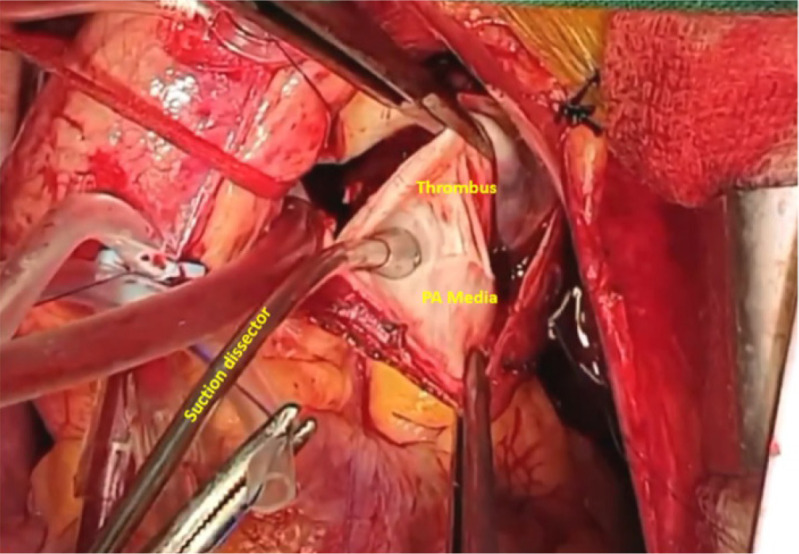
Delineation of the true plane of dissection in pulmonary thromboendarterectomy. PA=pulmonary artery.

Any fresh or loose clots are removed for visualization of the chronic thrombus. Thromboendoarterectomy then commences with creation of the correct plane in the media. The correct plane appears as a smooth pearly white structure which is amenable to peeling with controlled traction, countertraction, and a sweeping motion of the olive tipped sucker (Figure 4). The free cardiotomy sucker is employed to clear blood from the field. The surgeon should pay his/her undivided attention to the plane of dissection and should not shift his/her field of view for exchanging instruments.

**Fig. 5:**
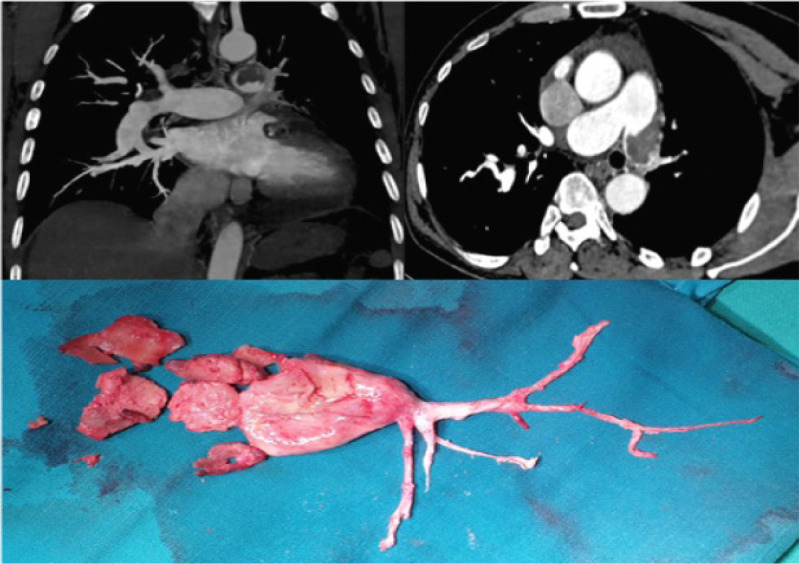
Computed tomography pulmonary angiogram and thromboendarterectomy specimen of patient 4.

Meanwhile, the blood collected from the cell saver is then transferred to the cardiotomy reservoir. In the endarterectomy of the sublobar branches, traction and dissection of the thrombus evert the media, and this may risk perforation of the PA. One should gently extract the thrombus which should come out spontaneously with its tapered tail (Figure 5).

After the endarterectomy, gentle positive pressure ventilation is initiated by the anesthetist to check for breach of PA wall which appears as air bubbles in the operating field. Brisk bright red back bleeding is an indirect sign of clearance from the endarterectomized segment.

For final confirmation, an endoscope (5 mm diameter, 0 degree) is inserted into the LPA and clearance is confirmed in each segment with the olive tipped sucker clearing the bronchial back bleed. The thrombus extracted is examined in toto to confirm complete PTE in all segments.

The RPA clamp is removed, and the incision is then extended from the vent insertion site to the right lower lobe branch just after the takeoff of the middle lobe artery. Visualization of the RPA tree is partially obscured due to the SVC running above it. Here, the modified mastoid retractor is applied between the SVC and aorta for better visualization of the pulmonary arterial tree. Alternatively, the SVC can also be retracted to the left for better viewing of the descending lobar branches. The dissection is carried out and the steps are analogous to the left as described.

Upon completion, the pulmonary arteriotomies are closed in two layers with 5-0 polypropylene sutures. The patient is then weaned off CPB; direct PA pressure manometry is done, and chest is closed after heparinization reversal and hemostasis.

## RESULTS

A total of four patients were operated for CTEPH from January 2019 to September 2020. Patient preoperative data is presented in Table 1. Preoperative transthoracic echocardiographic imaging revealed severe pulmonary hypertension with a median PASP of 85 mmHg, moderate tricuspid regurgitation (TR) in two patients, and severe TR in two patients. Severe RV dysfunction was found in all cases.

**Table 1:** Perioperative details of patients undergoing beating heart pulmonary thromboendarterectomy.

PREOPERATIVE FINDINGS					
	Patient 1	Patient 2	Patient 3	Patient 4	Median
Age (years)	23	24	46	47	35
Sex	Male	Male	Female	Male	
Preoperative ICU admission	-	-	-	-	
DVT	-	-	-	-	
NYHA class	III	IV	III	IV	
2D echo					
EF (%)	60	60	57	60	60%
PASP (mmHg)	72	68	98	120	85 mmHg
TR	Severe	Moderate	Severe	Moderate	
TAPSE (mm)	15	18	13	13	14 mm
Preoperative CTPA					
RPA, mm	16	*29	*21	35	
LPA, mm	*19	*26	*23	*33	
MPA, mm	*24	40	34	*39	
INTRAOPERATIVE FINDINGS					
Jamieson class	II	I	II	II (LPA only)	
UCSD surgical class	I	I	I	IC left	
CPB time (minutes)	108	117	135	161	126
Minimum temp (°C)	28	33.9	33.7	33	33.35
SpO_2_ (%)					
Preoperative	89	79	88	93	88.5
Postoperative	99	99	100	99	99
CVP					
Preoperative	25	22	19	26	23.5
Postoperative	13	12	12	11	12
ABP (systolic/diastolic/mean in mmHg)					
Preoperative	98/60/73	78/40/52	74/49/57	145/110/122	MAP 65
Postoperative	118/82/94	78/51/60	116/69/85	131/51/78	MAP 81.5
PAP (systolic/diastolic/mean in mmHg)					
Preoperative	65/34/44	51/48/49	24/15/18	104/49/67	mPAP 46.5
Postoperative	35/20/25	27/20/22	28/17/21	40/26/31	mPAP 23.5
POSTOPERATIVE FINDINGS					
Re-exploration	No	No	No	Yes	
Ventilation (hours)	24	15	12	96	19.5
ICU stay (days)	4	3	11	14	7.5
Hospital stay (days)	23	29	22	40	26
NYHA class	I	I	I	I	
2D echo					
EF (%)	58	60	60	65	60%
PASP (mmHg)	30	25	40	36	33 mmHg
TR	Trace	Trace	Moderate	Mild	
TAPSE (mm)	20	16	15	16	16 mm
Postoperative CTPA					
RPA, mm	18	19	17	24	
LPA, mm	22	10	20	18	
MPA, mm	28	35	33	34	
Follow-up (months)	15	14	4	2	9

* Thrombus 2D echo=2D echocardiography; ABP=arterial blood pressure; CPB=cardiopulmonary bypass; CTPA=computed tomography pulmonary angiogram; CVP=central venous pressure; DVT=deep venous thrombosis; EF=ejection fraction; ICU=intensive care unit; LPA=left pulmonary artery; MAP=mean arterial pressure (in mmHg); MPA=main pulmonary artery; mPAP=mean pulmonary artery pressure (in mmHg); NYHA=New York Heart Association Functional Classification; PAP=pulmonary artery pressure (by needle manometry); PASP=pulmonary artery systolic pressure; RPA=right pulmonary artery; SpO_2_=oxygen saturation; TAPSE=tricuspid annular plane systolic excursion; TR=tricuspid regurgitation; UCSD=University of California San Diego

Before PTE, intraoperative needle manometry revealed median preoperative PAP of 46.5 mmHg (Table 1). The thrombi removed were classified as Jamieson type II in three cases and type I in one case^[1]^. As per the UCSD surgical classification^[7]^, the thrombi were classified as class I in all cases with one case having complete occlusion of the LPA (IC left) (Table 1). Post PTE, needle manometry showed decreased median PAP of 23.5 mmHg (Table 1). The median CPB time was 126 minutes, and the median temperature was 33.35 °C (Table 1).

Patients postoperatively received mechanical ventilation for a median of 9.5 hours (Table 1). There was one re-exploration for bleeding, and the same patient had prolonged ventilation for 96 hours. The median intensive care unit stay was 7.5 days. There was no mortality. The median hospital stay was 26 days (Table 1).

Postoperative echocardiographic examination revealed decrease in median PASP (from 85 mmHg to 33 mmHg). Improvement in RV function was derived by TAPSE on 2D echo (median: 14 mm vs. 16 mm) (Table 1). Median postoperative SpO_2_ also showed improvement (88.5% vs. 99%). Postoperatively, over a follow-up ranging between 2-15 months, all patients reported improvement in their quality of life and were in NYHA class I (Table 1). There were no neurologic complications.

## DISCUSSION

PTE is the definitive treatment of CTEPH^[1]^. From its evolution of embolectomy to the current standard thromboendarterectomy, it cannot be over emphasized that complete clearance of the organized thrombus in the correct plane is vital for good outcomes^[8]^. Utley et al.^[9]^ emphasized that circulatory arrest was absolutely essential for distal segmental vessel thromboendarterectomy because of the increased bronchial circulation.

The use of DHCA and the adverse effects have been extensively described in literature for the conduct of PTE^[2-6]^. However, different strategies to avoid the side effects of DHCA, to obtain a relatively bloodless field, and to decrease operating time have been described.

In our procedure, we use four pump suckers and one external sucker connected to cell saver which helps us in obtaining a bloodless field. DHCA and its potential complications are thus avoided. Balloon catheter occlusion of the descending aorta to obtain a bloodless field as described by Hagl with the abovementioned setup was not needed^[10]^.

In our modifications for performing PTE on beating heart with CPB, we avoid division of the great vessels without compromising clearance, thereby minimizing vascular trauma^[9,10]^. As the procedure is conducted on beating heart with CPB support, we negate the need of complex cannulation of the aortic arch vessels for selective antegrade cerebral perfusion for PTE^[11-13]^. Our method of PTE differs from the method described by Mikus et al.^[14]^ in the fact that we use extensive venting of the left atrium via the RSPV, the right ventricle, the contralateral PA, and do not arrest the heart. We, however, follow a strategy of blood conservation similar to the described by the author by using a negative suction dissector connected to a cell saver. We also differ from the technique described by Bisoi et al.^[15]^ because we vent the RSPV and do not transect the SVC.

In our series, the hospital stay was prolonged because we are located in a remote area with poor patient compliance on follow-up. Our low-cost modifications for the conduct of PTE in this resource poor region are unique because of the use of mild hypothermia, the avoidance of cardioplegic arrest, avoidance of transection of the great vessels, and the ability to obtain a relatively bloodless field using external negative suction. The thorough checking of clearance the endarterectomized field by an endoscope also ensures that we can address remaining thrombus burden in the same setting.

### Limitations

This study has its limitations. First, it was a retrospective analysis covering a 20-month period. Second, small sample size is a limiting factor to gain significant results. However, we believe this study may provide some useful information for the surgical management of CTEPH patients

## CONCLUSION

The definitive treatment of chronic pulmonary hypertension is the complete evacuation of the thrombus load in the pulmonary vascular tree. Thromboendarterectomy under DHCA has been the most commonly technique employed to omit bronchial back bleed. Advances in perfusion, blood conservation, and our described modifications can make it possible to avoid circulatory arrest. However, the superiority of our technique cannot be established in view of the small number of cases.

**Table:** 

Authors' roles & responsibilities	
RLK	Substantial contributions to the conception or design of the work; or the acquisition, analysis, or interpretation of data for the work; drafting the work or revising it critically for important intellectual content; final approval of the version to be published
SR	Drafting the work or revising it critically for important intellectual content; agreement to be accountable for all aspects of the work in ensuring that questions related to the accuracy or integrity of any part of the work are appropriately investigated and resolved; final approval of the version to be published
MM	Agreement to be accountable for all aspects of the work in ensuring that questions related to the accuracy or integrity of any part of the work are appropriately investigated and resolved; final approval of the version to be published
MKS	Final approval of the version to be published
